# Newly Appointed Consultants

**Published:** 1968-10

**Authors:** 


### NEWLY APPOINTED CONSULTANTS

COLIN M. DAVIDSON, M.B., M.Ch., F.R.C.S. Ed., has been appointed Consultant Surgeon to the Frenchay-Cossham Group of Hospitals. He qualified at Glasgow University in 1951 and held Registrar appointments in Glasgow Royal Infirmary and the Western Infirmary, Glasgow. He visited the U.S.A. in 1959 on a University Scholarship and was awarded a Fellowship in Surgery at the Lahey Clinic where he extended his interest in colon and biliary surgery. Prior to his return, he visited a number of medical centres and clinics in North America. He was appointed Senior Surgical Registrar to Bristol in 1960 where he has worked since, apart from a two year secondment as Senior Lecturer in Surgery to the University of Khartoum in the Sudan. He has been elected a Councillor of the Lahey Clinic Alumni Association. His appointment to the Frenchay-Cossham Group of Hospitals will be maximum part-time.

Mr. H. J. ESPINER, M.B.Ch.B. (Otago) Ch.M. (Bristol) F.R.C.S., came to Bristol in 1960. He held a post as Research Associate in the University until 1963 when he took an appointment as Senior Registrar to the United Bristol Hospitals and the South Western Regional Board. He had previously worked at Christchurch, New Zealand, where he qualified in 1955. He is a General Surgeon with interests in abdominal surgery and cancer surgery. He is a keen high-handicap golfer. He is married with three children and lives in Stoke Bishop. His appointment will be maxiumum part-time.

Mr. A. H. YOUNG, B.D.S., F.D.S.R.C.S. (Eng. & Edin.), L.R.C.P., M.R.C.S., comes to Bristol from the Eastman Dental Hospital in London; he has previously worked at the Plastic and Jaw Surgery Unit at Mount Vernon Hospital, Northwood, and at Glasgow where he qualified in 1955. Apart from his main medical interests—oral surgery and oral diagnosis—he is interested in travel and photography. His appointment which also covers the Bath Clinical Area is whole-time.

